# Optimal outcome factors in maternity and newborn care for inpatient (hospital maternity ward-HMW) and outpatient deliveries (outhospital maternity clinics -OMC)

**DOI:** 10.1186/s12884-021-04319-x

**Published:** 2021-12-20

**Authors:** Azra Lukač, Nenad Šulović, Aleksandra Ilić, Milica Mijović, Dijana Tasić, Sonja Smiljić

**Affiliations:** 1 Community Health Center, Rožaje, Montenegro; 2 Department of Gynecology and Obstetrics, Faculty of Medicine, University in Priština, Kosovska Mitrovica, Serbia; 3 Institute of Preventive Medicine, Faculty of Medicine, University in Priština, Kosovska Mitrovica, Serbia; 4 Institute of Pathology, Faculty of Medicine, University in Priština, Kosovska Mitrovica, Serbia; 5 Clinic of Gynecology and Obstetrics “Narodni Front”, Belgrade, Serbia; 6 Institute of Physiology, Faculty of Medicine, University in Priština, Kosovska Mitrovica, Serbia

**Keywords:** OI score, Inpatient maternity ward, Outpatient maternity clinic

## Abstract

**Background and objectives:**

The aim of the study was to use the United States Optimality Index (OI-US) to assess the feasibility of its application in making decisions for more optimal methods of delivery and for more optimal postpartum and neonatal outcomes. Numerous worldwide associations support the option of women giving birth at maternity outpatient clinics and also at home. What ought to be met is the assessments of requirements and what could be characterized as the birth potential constitute the basis for making the right decision regarding childbirth.

**Materials and methods:**

The study is based on a prospective follow-up of pregnant women and new mothers (100 participants) who were monitored and gave birth at the hospital maternity ward (HMW**)** and pregnant women and new mothers (100 participants) who were monitored and gave birth at the outhospital maternity clinics **(**OMC). Selected patients were classified according to the criteria of low and medium-risk and each of the parameters of the OI and the total OI were compared.

**Results:**

The results of this study confirm the benefits of intrapartum and neonatal outcome, when delivery was carried out in an outpatient setting. The median OI of intrapartum components was significantly higher in the outpatient setting compared to the hospital maternity ward (97 range from 24 to 100 vs 91 range from 3 to 100). The median OI of neonatal components was significantly higher in the outpatient compared to the inpatient delivery. (99 range from 97 to 100 vs 96 range from 74 to 100). Certain components from the intrapartum and neonatal period highly contribute to the significantly better total OI in the outpatient conditions in relation to hospital conditions.

**Conclusion:**

Outpatient care and delivery provide multiple benefits for both the mother and the newborn.

## Introduction

More and more women find it difficult to come to terms with involuntary hospitalization for the sole purpose of childbirth [1]. This argument stands from the belief that, after a normal pregnancy, there is no need for excessive medicalization for something as natural as childbirth. Numerous worldwide associations support the option of women giving birth at maternity outpatient clinics and also at home [2]. At the same time, even after a normal pregnancy, doctors call for caution and cannot guarantee (in advance) the safety of the child and the mother. The assessments of requirements that need to be met and what could be characterized as the birth potential constitute the basis for making the right decision regarding childbirth and represent the beginning of proper medical care.

What we would define as a risk factor regarding the delivery itself, the delivery setting, types of delivery and those in charge of medical care has dominated the decision-making process for decades [2, 3]. While most deliveries take place in hospital clinics or maternity wards, a small percentage (1.8% nationally) take place in outpatient clinics with the assistance of midwives [4].

The very act of giving birth in a hospital has lost some of its humanity. Women are most often half- asleep during childbirth since, in most cases, the pain is relieved by pharmaceuticals [1].

Pain relief during labor is of course desirable, but sometimes the side effects cannot be avoided. The improvement was made by introducing epidural analgesia as a method of pain control, but this method is not widely used in obstetric practice as a standard regimen. The mother usually feels abandoned, surrounded by unfamiliar people performing procedures unknown to her. These factors cause stress that affects the course of labor, slowing it down and causing a cascade of intervention [5, 6].

### This concept has several drawbacks

It allows for a natural physiological event to be turned into a medical procedure.

It restricts a woman’s freedom to experience the birth of her children in her own way, in the environment she previously chose.

It involves unnecessary interventions; requires a well-equipped hospital and includes all accompanying costs [7].

Considering these factors, delivery in outpatient conditions, primarily outpatient maternity clinics as well as domestic obstetrics, are gaining in popularity. The atmosphere is more personal, more humane, trust in the medical staff is greater and cooperation between the mother and the staff is better. It also offers the possibility for the father (husband) and other family members to accompany the mother during childbirth. All these facts contribute to the satisfaction and happiness of bringing offspring to the family. However, this practice is not equally represented in the world. The Scandinavian countries, the Netherlands and North America, went the furthest in its implementation; where between 15 and 30% of births are performed in outpatient conditions [8]. There are five outpatient maternity clinics in Montenegro, of which the outpatient maternity clinic in Rožaje is the largest.

In order to prepare and acquire the best conditions for vaginal birth, monitoring, preparation and evaluation and finally the decision on how to complete the birth process requires exceptional commitment to the patient [1]. Pregnancy does not qualify as a pathological category, but it is a physiologically altered condition, and in order for that period to pass as purposefully as possible, increased control of the condition of both the pregnant woman and the fetus is needed, as well as the absolute cooperation and trust between the patient and the obstetrician. For practical reasons, for the purpose of monitoring and controlling a pregnancy we use general rules, protocols, algorithms, questionnaires and indices which significantly facilitate this work.

The optimality index (OI) should be the basis on which the patient’s condition before and during pregnancy, during and after childbirth as well as the condition of the fetus and the neonate are based. It was created in the USA the 1990s, and was modified in 2001 and 2006, as it is still used today with an update every 5 years [9]. It is mostly used in the USA, Great Britain and the Scandinavian countries. It implies and cultivates the concept of optimality, which is different from the state of normalcy [10].

It is based on the maxim “maximum score with minimum intervention” [11]. Turkey and the Netherlands have adjusted the optimality index used in the USA (OI-US) and the results of their studies have shown that OI-TR and OI-NL, adapted to the maternity protection system in these countries, are valid and reliable for assessing maternity care [12, 13].

The study aimed to use the United States Optimality Index (OI-US) to assess the feasibility of its application in making decisions for a more optimal way of delivery and more optimal postpartum and neonatal outcomes.

## Materials and methods

This study is based on prospective monitoring of pregnant women and mothers who were monitored and delivered in the outpatient maternity clinic in Rožaje (100 in total) and pregnant women and mothers who were monitored and delivered at the maternity ward of the General Hospital in Berane (100 in total).

Annually, between 130 and 150 births are performed at the outpatient maternity clinic in Rožaje, which is the largest of the five existing such clinics in Montenegro, and about 950 births at the General Hospital in Berane.

### Period of conducting examinations/research: 2019–2020

Each patient signed a consent form, which outlined the reasons, method and manner of conducting the examinations and research. The heads of the examining institutions at General Hospital-Berane, Health Center with outpatient maternity clinic - Rozaje then granted written consent.

In order to ensure uniformity among the groups, the selected patients were classified according to certain criteria.

**The basic criteria for inclusion in the study are:**

- Age (between 20 and 40)
- Multiple births (no more than 3 pregnancies)
- Pregnant women without pre-recorded comorbiditу

**Exclusion criteria:**

- First birth
- Previous caesarean section or any operation involving a uterine scar (myomectomy, metroplasty or adenomyosis)
- Multifetal pregnancy
- More than 3 births
- Women whose pregnancies were the result in vitro fertilization

### Statistical data processing

Each OI parameter (modified OI that included current pregnancies and childbirth) was evaluated as optimal or not. The obtained results for each parameter are expressed as a frequencies and percentage for each of the four components of OI-US: antepartum (12 items), intrapartum (19 items), neonatal (9 items) and postpartum (8 items) as well as the total Optimality Index (48 items).

Measures of descriptive statistics were used to describe the parameters of importance, depending on their nature: frequencies, percentages, medians and range. The Shapiro-Wilk test was used to check the normality of the distribution. In addition to descriptive statistics, the chi-square test, the Fisher test, the Mann-Whitney U-test, and the Median test were used to determine the differences between groups.

Multiple logistic regression determined the model where the dependent variable was the place of delivery (outpatient/inpatient). Statistical hypotheses were tested at the level of statistical significance of 0.05. The SPSS Statistics 22 program (SPSS Inc., Chicago, IL, USA) was used for statistical data processing.

### Patient and public involvement

A questionnaire explained to the patient the type of research, the goal, manner and its purpose. Patient privacy was also guaranteed and the right of the patient to cancel the research No randomized controlled trials took place, results were compared from the classified groups, as explained in the project protocol.

## Results

Research results were derived from a sample of 200 pregnant women between the ages of 20 and 40. The mean age of the pregnant women was 30.5 ± 5.4. The age of the pregnant women who gave birth out of a hospital was 30.8 ± 6.0. The median was 30.0 (range 20–40), the mean age of women delivered in the hospital was 30.2 ± 4.8, median 30.0 (range 20–40). The age of pregnant women did not differ significantly according to the place of delivery, *p* = 0.523. Results were observed through antepartum, intrapartum, neonatal and postpartum components.

The socio-demographic parameters assessed according to the OI-US were marital status, use of cigarettes, alcohol and drugs. All pregnant women were married, none of them used alcohol and drugs and 19% of pregnant women with out-of-hospital delivery and 20% of them in hospital delivery used cigarettes, which is not a statistically significant difference (0.985).

### Antepartum components

Bleeding in the II and III trimesters of pregnancy was a significant feature in pregnant women who gave birth in hospital conditions (5 cases), while there were no cases in outpatient deliveries (*p* = 0.024).

Serious antepartum complications that were not recorded during ongoing pregnancies in subject women were: gestational diabetes, mental illness and Rh sensitization. All other complications characteristic at this stage of pregnancy were represented with an optimal frequency that did not differ significantly in relation to the place of delivery, in outpatient or inpatient settings (Table 1).

**Table 1:** Items available for analysis for antepartum section of the OI (*n* = 100)

Antepartum items	OMW		HMW		*p*
	Optimal	Not optimal	Optimal	Not optimal	
	%	%	%	%	
Anemia	88	12	82	18	0.235
Gestational diabetes	100	0	100	0	/
Mental disorders	100	0	100	0	/
Placenta previa	100	0	99	1	1.00
Preeclampsia	100	0	99	1	1.00
Pyelonephritis	99	1	99	1	1.00
Rh sensitization	100	0	100	0	/
Bleeding in II or III trimesters	100	0	95	5	0.024*
Adequate antenatal care	50	50	49	51	0.888
Amniocentesis	97	3	100	0	0.081
Use of medication	89	11	84	16	0.301
NST and BFP	100	0	100	0	/

*BFT* biophysical fetal profile

*significant *p* < 0.05 NST- non stress test or prenatal non stress test

**Fig. 1:**
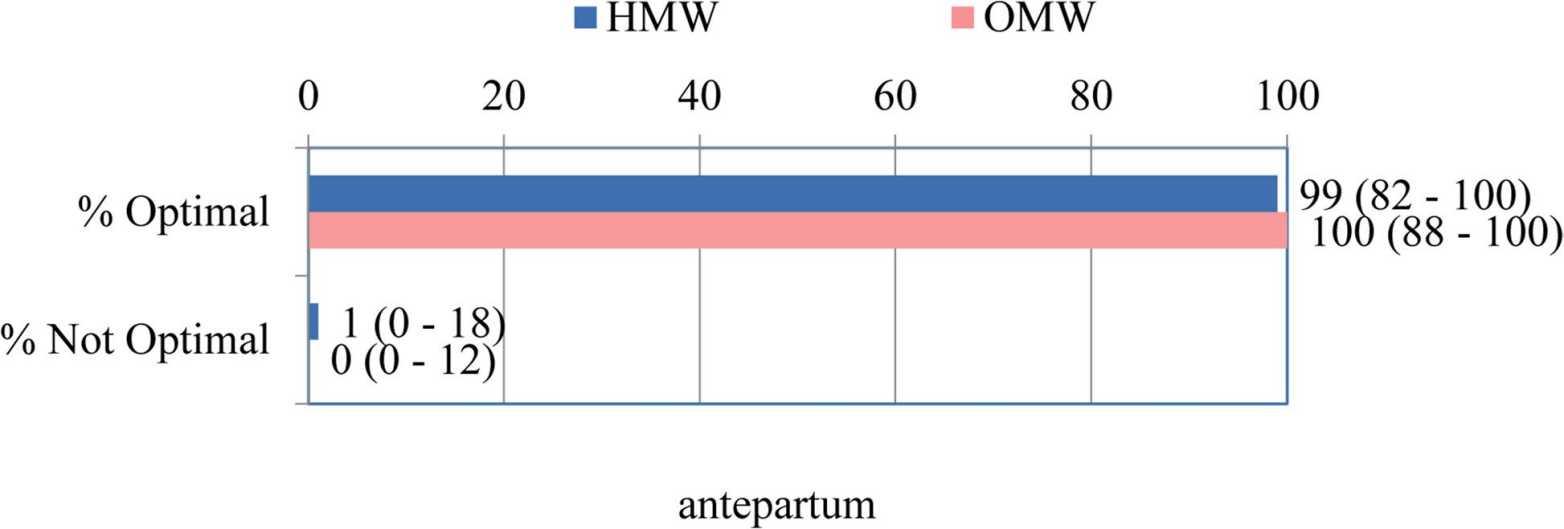
The total OI score for antepartum section – MEDIAN (range)

The previous distribution of antepartum characteristics determined that the median of the total Optimality Index of this component did not differ significantly in relation to the compared groups of pregnant women who were delivered in outpatient or hospital conditions (100 vs 99, *p* = 0.289) (Fig. 1).

### Intrapartum components

Many parameters within the intrapartum period differed significantly depending on the place of delivery (outpatient/inpatient). In hospital conditions, during phase III of childbirth, there was a significantly higher frequency of amniotomy, cesarean section, induction and stimulation of childbirth, application of painkillers and medication other than oxytocin, which are all considered suboptimal characteristics of childbirth. In addition, there were other, less optimal measures taken during childbirth, grade III or IV episiotomy or grade I or II lacerations, fetal heart rate abnormalities, postpartum hemorrhage and blood transfusions that were more appropriate for hospital conditions as suboptimal events.

In the conditions of outpatient preparation and childbirth, according to the global obstetric assessment, epidural analgesia, perineum lacerations which require sutures, and cervical lacerations were suboptimal and used with higher frequency. The presence of a support person during childbirth was a favorable feature of outpatient childbirth conditions that was quantified as a significant frequency relative to hospital births. Other intrapartum components did not differ significantly in relation to the delivery setting (Table 2).

**Table 2:** Items available for analysis for intrapartum section of the OI (n = 100)

Intrapartum items	OMW		HMW		*p*
	Optimal	Not optimal	Optimal	Not optimal	
	%	%	%	%	
The period between the rupture of the membrane and birth, < 24 h	100	0	100	0	/
Amniotomy	86	14	49	51	< 0.001*
Clear amniotic fluid	97	3	91	9	0.074
Induction or stimulation of labor	92	8	55	45	< 0.001*
Use of analgesics for labor	88	12	55	45	< 0.001*
Epidural analgesia	90	10	97	3	0.045*
Fetal heart rate abnormalities^a^	100	0	92	8	0.004*
Presence of a support person during labor	24	76	7	93	< 0.001*
Childbirth conditions^b^	100	0	95	5	0.024*
Fetus position (cephalic)	96	4	95	5	0.733
Cesarean section	100	0	73	27	< 0.001*
Episiotomy	95	5	98	2	0.248
Perineal laceration requiring sutures and cervical lacerations	86	14	98	2	0.002*
Grade III or IV episiotomy or grade I or II lacerations	100	0	92	8	0.004*
Medication other than oxytocin during stage III of childbirth	97	3	71	29	< 0.001*
Skin to skin contact	98	2	72	28	< 0.001*
Placental retention longer than 30 min	99	1	97	3	0.312
Postpartum haemorrhage	99	1	88	12	< 0.002*
Blood transfusion	100	0	91	9	< 0.002*

*significant *p* < 0.05

^a^CTG, Doppler, fetoscopy, CTG finding (non – reactive)

^b^Unprompted pushing, Postpartum delivery, Instrumental vaginal delivery

**Fig. 2:**
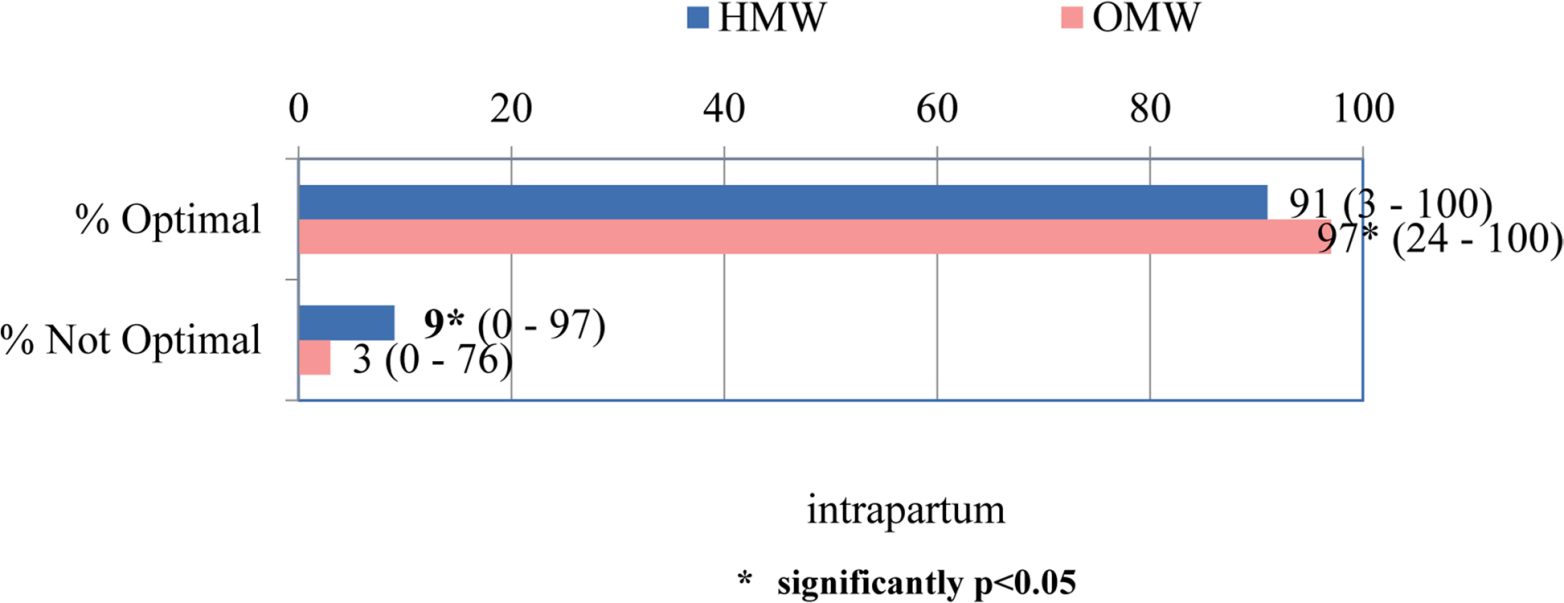
The total OI score for intrapartum section – MEDIAN (range)

The frequency distribution of previously analyzed intrapartum components determined the median of optimal conditions that was significantly higher in outpatient than in hospital delivery settings (97% vs 91% of cases). At the same time, the frequency of suboptimal components was significantly higher in outpatient compared to hospital delivery conditions (*p* = 0.01; 0.05) (Fig. 2).

### Neonatal components

Individually and within neonatal components, the greatest statistical significance belongs to the role of optimal lactation at the time of hospital discharge (up to 72 h after delivery) in outpatient maternity clinics in relation to hospital maternity wards. This is followed by a higher frequency of optimal neonatal intensive care, then the absence of bacterial infections, as well as the optimal assessment of gestational age, all of which were, in a significantly higher number of cases, appropriate for the outpatient delivery setting. Other neonatal components (infant birth weight, APGAR score at 5 min, congenital anomalies, respiratory distress syndrome), shown in Table 3, did not differ significantly with respect to the delivery setting.

**Table 3:** Items available for analysis for neonatal section of the OI (*n* = 100)

Neonatal items	OMW		HMW		*p*
	Optimal	Not optimal	Optimal	Not optimal	
	%	%	%	%	
Estimation of gestational age (37-42 weeks)	99	1	93	7	0.030*
Infant birth weight (2500-4000 g)	98	2	96	4	0.407
APGAR 5 min (7,8,9,10)	99	1	97	3	0.312
Neonatal intensive care	98	2	88	12	0.006*
Congenital anomalies	100	0	98	2	0.115*
Bacterial infections other than sepsis	100	0	93	7	< 0.007*
Respiratory distress syndrome	99	1	98	2	0.567
Other complications including sepsis	100	0	100	0	/
Lactation at the time discharge (up to 72 h after delivery)	97	3	74	26	< 0.001*

*significant *p* < 0.05

**Fig. 3:**
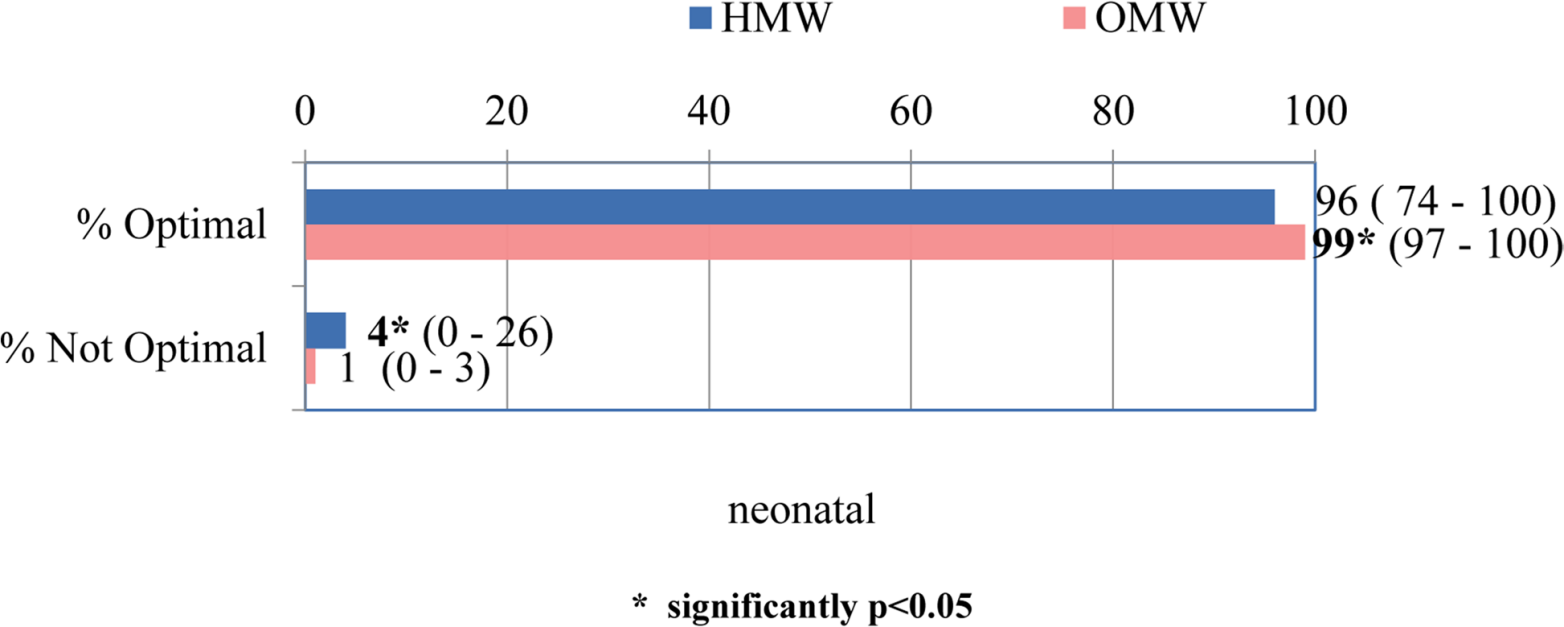
The total OI score for neonatal section MEDIAN (range)

The median Optimality Index of neonatal components was significantly higher in outpatient compared to inpatient deliveries. At the same time, the median of suboptimal indices was higher in hospital conditions compared to outpatient ones. (*p* = 0.021, Fig. 3.).

### Postpartum components

Local suture infection did not occur in any of the cases of outpatient deliveries during the postpartum period, which is a significant difference compared to six (suboptimal) events in hospital settings (*p* = 0.013). At this period, the frequency of prescribed medication in maternity wards was significantly higher in relation to outpatient maternity clinics (15 to 5 cases).

Other postpartum components (perinatal death between the birth and 72 h of life, cystitis, endometritis) were not observed in outpatient settings. Yet, this is not a significant difference in relation to their prevalence (1-2 cases) in outpatient maternity clinics.

Maternal death, hematoma and mastitis did not occur in any of the cases, regardless of the delivery setting (Table 4).

**Table 4:** Items available for analysis for postpartum section of the OI (*n* = 100)

*Postpartum items*	OMW		HMW		*p*
	Optimal	Not optimal	Optimal	Not optimal	
	%	%	%	%	
Perinatal death occurring between birth and 72 h	100	0	99	1	1.00
Cystitis	100	0	99	1	1.00
Endometritis	100	0	98	2	0.155
Hematoma	100	0	100	0	/
Local suture infection	100	0	94	6	0.013*
Mastitis	95	5	95	5	1.00
Prescribed medication	95	5	85	15	0.018*
Maternal death	100	0	100	0	/

*significant *p* < 0.05

**Fig. 4:**
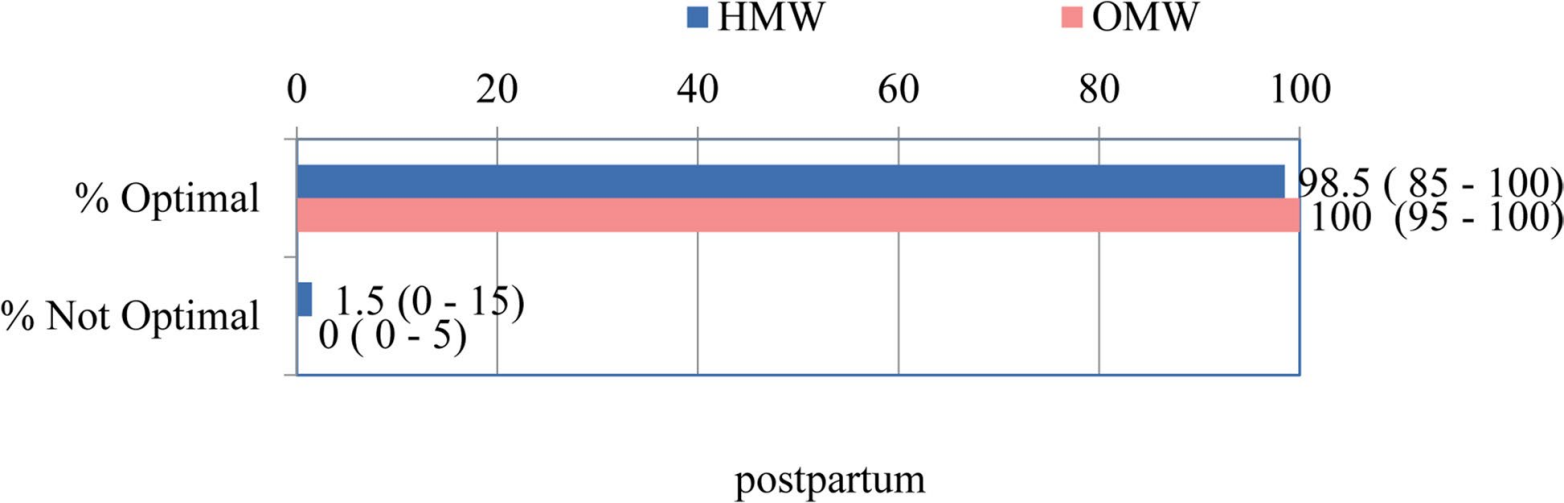
The total OI score for postpartum section MEDIAN (range)

The median Optimality Index for previously analyzed postpartum components, was at the border of statistically significant differences in relation to the place of delivery: outpatient or inpatient (100% vs 98.5%, *p* = 0.050) (Fig. 4).

**Fig. 5:**
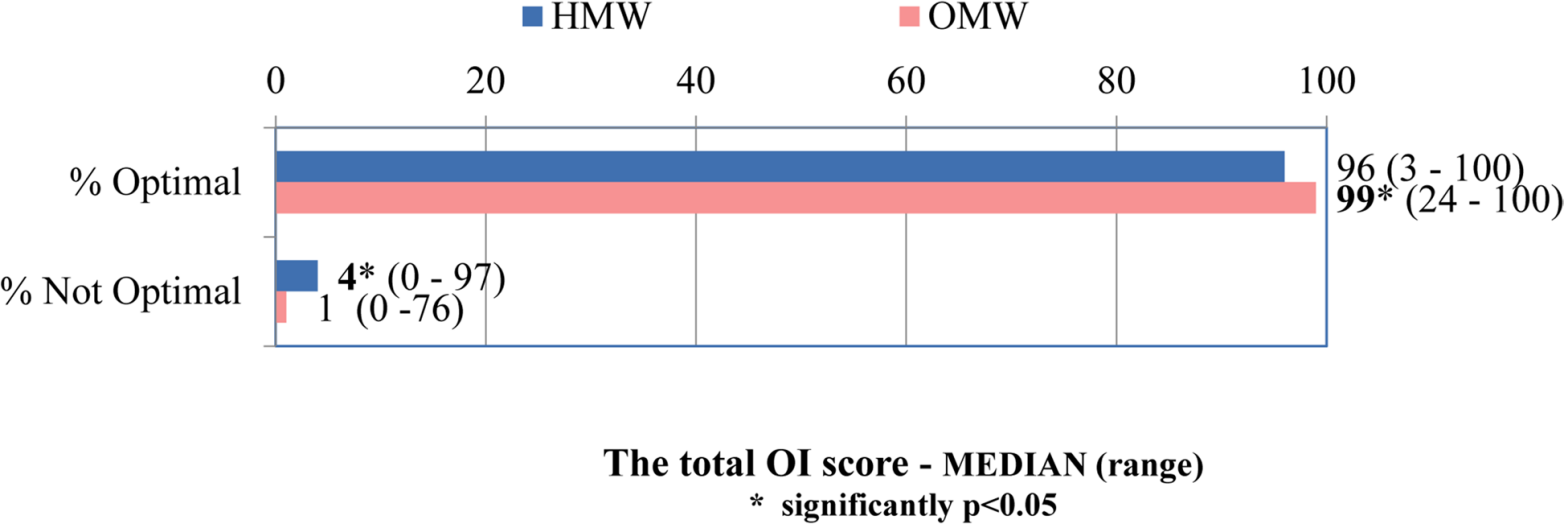
The total OI score MEDIAN (range)

The Total Optimality Index (OI) contained all of the synthesized, previously analyzed parameters of individual components (48 items). For the considered sample of subjects, the median total optimality index (which included antepartum, intrapartum, neonatal and postpartum components) was in the 99% range (24 -100%) for the outpatient maternity clinics as opposed to the hospital maternity wards where it was in the 96% range (3 -100%). Statistical testing of the significance of differences shows that this difference is significant, at the adopted level of reliability, and favors the optimality of delivery conditions in outpatient settings. (*p* = 0.001, Fig. 5).

### Logistic regression analysis

All variables that stood out as statistically significant for the alpha level of 0.001 in the primary analysis were inserted into the logistic regression model where delivery settings (outpatient/inpatient conditions) represented the dependent variable (Table 5).

**Table 5:** Logistic regression

Independent variables	Multiple logistic regression		
	B	*p*	OR (95%CI)
Amniotomy	1.39	0.003*	4.01 (1.63-9.91)
Induction or stimulation of labor	18.88	0.997	157,556,047.7 (0.0−/)
Use of painkillers	−18.19	0.998	0.0 (0.0-0.0)
The presence of a person of support during childbirth	−1.53	0.007*	0.22 (0.07-0.66)
Cesarean section	35.356	0.997	2.2e ^15^ (0.0−/)
Medication other than oxytocin during stage III of childbirth	−0.29	0.779	0.75 (0.10-5.51)
Skin to skin contact	0.03	0.980	1.03 (0.09-11.83)
Lactation at the time discharge (up to 72 h after delivery)	−1.24	0.082	0.29 (0.07-1.17)

The model contains 8 independent variables and the whole model was statistically significant (chi-square = 84.877, *p* < 0.001), Table 5.

In the multiple logistic regression model, amniotomy was singled out as a statistically significant factor in delivery in hospital conditions (B = 1.39; *p* = 0.003), and pregnant women who underwent amniotomy four times were more likely to give birth in hospital conditions. However, the presence of a support person during childbirth is a significant factor that increases the chance of pregnant women opting for delivery in outpatient settings (B = − 1.53; *p* = 0.003).

Another distinguishing factor is lactation at the time of hospital discharge (up to 72 h after delivery), which is closer to the statistical significance than other variables in the model and indicates that the successful onset of lactation before discharge is another factor in favor of outpatient delivery (B = − 1.24; *p* = 0.082).

## Discussion

The main goal of the presented study was to quantify and compare the optimal outcome factors in maternity and neonatal care in inpatient and outpatient deliveries. The aspiration was to achieve the optimal conditions of pregnant women and mothers that would guarantee adequate prerequisites for physiological childbirth. Maternity care models have a basis in the scientific literature that shows the existing relationship between the quality of care and the data on the related support. However, the outcome commonly presented in the maternity support data is usually only limited to the presence of morbidity and mortality in women and their newborns. In that sense, the full range of childbirth experience is not included, nor is the medical support that would promote optimal care conditions, which is often a consequence of the lack of an appropriate measuring system for this type of care [14].

The Optimality Index used as a measuring instrument in this study included only one part of the original OI-US score. Namely the first part, the Perinatal Background Index PBI (demographic, medical, and obstetric history factors) is not presented in our paper since the attention was placed on the Optimality Index.

The Optimality Index (OI) is a combined measure of antepartum, intrapartum, neonatal, and postpartum care practices and health outcomes. Over time, the concept of optimality and its components has undergone numerous changes and, if necessary, reductions [9, 15, 16].

The advantages of different versions of outpatient delivery conditions have been noticeably considered with reference to several varieties of beneficial effects of such an approach. Among the many observed benefits, we can distinguish an increase in the probability of vaginal births, a lower rate of interventions, including epidural anesthesia, and a shorter labor duration [17–22].

The total Optimality Index in this paper is very high in outpatient maternity clinics (median 99% range 24-100%), but also in hospital maternity wards (96% range 3-100%). For the considered sample of subjects, the median of the Total Optimality Index (which included antepartum, intrapartum, neonatal and postpartum components) was significantly higher in the outpatient maternity clinics.

The results of other authors also show significantly high medians or average scores of optimality indices (total or by components). Numerous factors limit the direct comparison of our results with other studies. This implies the differences in design and methods, with respect for randomization, analysis in accordance with the intended and current place of delivery, present risk and similar. Compared to most studies of this subject matter, the concept of low-risk pregnancies was quite restrictive. Thus, the exclusion criteria in the study included many characteristics that were accepted in other studies. Comparisons of other research results often included the division of pregnant women by parity, by place of delivery (rural-urban environment), whether caesarean section occurred or not, whether epidural was administered or not and some other delivery conditions [14, 19, 23, 24].

To observe the outcome of deliveries and the quality of care in the appropriate conditions through the parameters of morbidity/mortality of mothers and newborns would require many years of research with a huge amount of analyzed data (meta-analyzes) since, fortunately, these occurrences are not frequent [22, 25].

By comparing the obtained optimality indices by components, we noticed that the significant differences in relation to the place of care and child delivery occur mostly during the intrapartum and then in the neonatal period. The median optimality index of intrapartum components was significantly higher in the outpatient delivery settings compared to the inpatient maternity ward (97 range from 24 to 100 vs 91 range from 7 to 100). In hospital conditions, the use of caesarean section as a mode of delivery was noticeable, as well as the application of other physically invasive measures during delivery, such as the significantly higher frequency of amniotomy, induction and stimulation of childbirth, application of painkillers, all of which are considered suboptimal characteristics of the course of child delivery. The higher frequency of interventions during childbirth and oxytocin usage was confirmed by a study conducted in Australia between 2000 and 2012 [25]. Women who plan to give birth in outpatient maternity clinics are twice as likely to have a vaginal birth compared to women who plan to give birth in hospitals [24].

In all comparative analyzes, the rate of cesarean deliveries occurring in hospital conditions has been proven to be significantly higher compared to alternative delivery settings that included private clinics and home deliveries with adequate assistance from midwives or other medical staff [26–30]. However, there may be some limitations in such analyzes, so the possibility of direct comparison must be considered within the conditions in which the delivery took place and taking into account many factors, including parity, epidural analgesia, oxytocin administration, transfer rate, medical staff proficiency and similar [22]. Therefore, the approach to such comparisons requires the application of much more rigorous descriptions of conditions [25].

Back in 2002, a research was conducted in the US by The Maternity Center Association (MCA) with the aim of pointing out that a technologically-invasive childbirth is a significant experience for most women in the US, with increasing tendency to promote technological benefits to support best pregnancy health outcomes [23]. It has been proven that, even though many practices are not effectively supported by the scientific literature, they are routinely applied during perinatal care in healthy women. Following the findings of previous and similar research, group organizations have been formed, advocating for a less technological approach to maternity care and calling for the application of less technologically oriented practices and increasing the number of options for pregnant women [31, 32].

Contrary to many research results, in this study, laceration as an intrapartum component had a significant frequency in outpatient deliveries, which coincides with the results of the Dutch birth center study, obtained using OI-NL2015, especially in nulliparous women [33]. There are reasons described in the literature that could be responsible for this suboptimal component. Higher degree of perinatal laceration includes obstetric and neonatal factors and characteristics of the newborn. Possible factors include mothers’ higher body weight, and often related newborns’ higher body weight, but also the application of instrumental vaginal births [34].

The presence of a support person during childbirth was proven to be a favorable feature of outpatient delivery but also, as proven through a logistic regression analysis, a factor that increases the chance of pregnant women opting for outpatient delivery. Most analyzes of perinatal conditions from the literature confirm the importance of this factor in the intrapartum process [19, 20].

In the neonatal period, the most important optimality component is lactation at the time of hospital discharge (up to 72 h after giving birth), followed by a significantly less suboptimal presence of bacterial infections and optimal gestational age.

The application of logistic regression analysis also confirmed that certain components from the intrapartum and neonatal period contribute the most to the significantly better total optimality index in outpatient conditions compared to hospital conditions.

Our research operationalized this approach, with enough perspective data to satisfy the sample size calculations. This study offers a broad overview of relevant factors in contemplating the physiology of childbirth in the outpatient delivery approach in Montenegro, including the assessment of validity, and is the first such study in the Balkans. This was also the first time the OI was used in Montenegro to support physiological childbirth. The design of the study was prospective, which made it possible to monitor the pregnancy of each pregnant woman from the beginning of gestation to delivery. Since only one outpatient maternity clinic is involved, this can be considered a disadvantage of this study.

## Conclusion

Outpatient care and delivery provide multiple benefits for both the mother and the newborn. The results of this study, based on the comparison of basic components and the total Optimality Index, confirm the benefits of intrapartum and neonatal outcome, when delivery was performed in an outpatient setting. Also, the total Optimality Index has a better score in outpatient compared to inpatient conditions.

## Data Availability

The data presented in this study are available on reasonable request from the corresponding author.

## References

[1] Simić J, Jerinić J (2014). Pravo na porođaj kod kuće– izbor, a ne slučajnost i neke pravne posledice. Pravni Zapisi.

[2] Johanson R, Newburn M, Macfarlane A. Has the medicalisation of childbirth gone too far? BMJ. 2002;324(7342):892–5. 10.1136/bmj.324.7342.892. 10.1136/bmj.324.7342.892 PMC1122835 11950741

[3] UNFPA, ICM, WHO (2011). State of the world’s midwifery 2011: delivering health, saving lives.

[4] Australian Institute of Health and Welfare (2017). Australia’s mothers and babies 2015—in brief. Perinatal statistics series no. 33. Cat no. PER 91.

[5] Hodnett ED, Gates S, Hofmeyr GJ, Carol SC (2013). Continuous support for women during childbirth. Cochrane Database Syst Rev.

[6] Green J, Amis D, Hotelling BA (2007). Care practice 3. Continuous labor support. J Perinat Educ.

[7] Tunçalp Ö, Were WM, MacLennan C, Oladapo OT, Gülmezoglu AM, Bahl R (2015). Quality of care for pregnant women and newborns – the WHO vision. BJOG..

[8] Hendrix M, Van Horck M, Moreta D, Nieman F, Nieuwenhuijze M, Severens J, Nijhuis J (2009). Why women do not accept randomisation for place of birth: feasibility of a RCT in the Netherlands. BJOG: an international. J Obstet Gynaecol.

[9] Murphy PA, Fullerton JT (2006). Development of the optimality index as a new approach to evaluating outcomes of maternity care. J Obstet Gynecol Neonatal Nurs.

[10] Kennedy H (2006). A concept analysis of optimality in perinatal health. J Obstet Gynecol Neonatal Nurs.

[11] Wiegers TA, Keirse MJ, Berghs GA, van der Zee J (1996). An approach to measuring quality of midwifery care. J Clin Epidemiol.

[12] Yucel C, Taskin L, Low LK (2015). Validity and reliability of the Turkish version of the optimality index-US (OI-US) to assess maternity care outcomes. Midwifery..

[13] Thompson SM, Nieuwenhuijze MJ, Budé L, de Vries R, Low LK (2018). Creating an optimality index - Netherlands: a validation study. BMC Pregnancy Childbirth.

[14] Murphy PA, Fullerton JT (2001). Measuring outcomes of midwifery care: development of an instrument to assess optimality. J Midwifery Womens Health.

[15] Kyllerman M, Hagberg G (1983). Reduced optimality in pre- and perinatal conditions in a Swedish newborn population. Neuropediatrics.

[16] Touwen B, Huisjes H, Jurgens-van der Zee AD, Bierman-van Eendenburg M, Smrkovsky M, Olinga A (1980). Obstetrical condition and neonatal neurological morbidity. An analysis with the help of the optimality concept. Early Hum Dev.

[17] World Health Organization (2018). WHO recommendations: intrapartum care for a positive childbirth experience.

[18] Oladapo OT, Tunçalp Ö, Bonet M, Lawrie TA, Portela A, Downe S (2018). WHO model of intrapartum care for a positive childbirth experience: transforming care of women and babies for improved health and wellbeing. BJOG..

[19] Bohren MA, Hofmeyr GJ, Sakala C, Fukuzawa RK, Cuthbert A (2017). Continuous support for women during childbirth. Cochrane Database Syst Rev.

[20] Sandall J, Soltani H, Gates S, Shennan A, Devane D (2016). Midwife-led continuity models versus other models of care for childbearing women. Cochrane Database Syst Rev.

[21] McLachlan HL, Forster DA, Davey MA, Farrell T, Gold L, Biro MA (2012). Effects of continuity of care by a primary midwife (caseload midwifery) on caesarean section rates in women of low obstetric risk: the COSMOS randomised controlled trial. BJOG..

[22] Brocklehurst P, Hardy P, Hollowell J, Linsell L, Macfarlane A, McCourt C (2011). Perinatal and maternal outcomes by planned place of birth for healthy women with low risk pregnancies: the birthplace in England national prospective cohort study: birthplace in England collaborative group. BMJ..

[23] Low LK, Miller J (2006). A clinical evaluation of evidence- based maternity care using the optimality index. J Obstet Gynecol Neonatal Nurs.

[24] Nethery E, Gordon W, Bovbjerg ML (2018). Rural community birth: maternal and neonatal outcomes for planned community births among rural women in the United States, 2004–2009. Birth..

[25] Scarf VL, Rossiter C, Vedam S, Dahlen HG, Ellwood D, Forster D (2018). Maternal and perinatal outcomes by planned place of birth among women with low-risk pregnancies in high-income countries: a systematic review and meta-analysis. Midwifery..

[26] Homer CSE, Cheah SL, Rossiter C, Dahlen HG, Ellwood D, Foureur MJ (2019). Maternal and perinatal outcomes by planned place of birth in Australia 2000–2012: a linked population data study. BMJ Open.

[27] Scupholme A, McLeod AG, Robertson EG (1986). A birth center affiliated with the tertiary care center: comparison of outcome. Obstet Gynecol.

[28] Jackson DJ, Lang JM, Swartz WH, Ganiats TG, Fullerton J, Ecker J (2003). Outcomes, safety, and resource utilization in ac ollaborativecare birth center program compared with traditiona lphysician- based perinatal care. Am J Public Health.

[29] Feldman E, Hurst M (1987). Outcomes and procedures in low risk birth: a comparison of hospital and birthcenter settings. Birth.

[30] David M, vonSchwarzenfeld HK, Dimer J, Kentenich H (1999). Perinatal outcome in hospital and birth center obstetric care. Int J Gynaecol Obstet.

[31] The Mother-Friendly Childbirth Initiative (1996). The first consensus initiative of the Coalition for Improving Maternity Services (CIMS). Birth Gaz.

[32] Sakala C, Gyte G, Henderson S, Neilson JP, Horey D (2001). Consumer-professional partnership to improve research: the experience of the Cochrane Collaboration’s Pregnancy and Childbirth Group. Birth..

[33] Hermus MAA, Hitzert M, Boesveld IC, van den Akker-van Marle ME, van Dommelen P, Franx A (2017). Differences in optimality index between planned place of birth in a birth centre and alternative planned places of birth, a nationwide prospective cohort study in The Netherlands: results of the Dutch Birth Centre Study. BMJ Open.

[34] Merz WM, Tascon-Padron L, Puth M-T, Heep A, Tietjen SL, Schmid M, Gembruch U (2020). Maternal and neonatal outcome of births planned in alongside midwifery units: a cohort study from a tertiary center in Germany. BMC Pregnancy Childbirth.

